# A Novel Finding of an HLA Allele’s and a Haplotype’s Relationship with SARS-CoV-2 Vaccine-Associated Subacute Thyroiditis

**DOI:** 10.3390/vaccines10121986

**Published:** 2022-11-23

**Authors:** Melisa Sahin Tekin, Goknur Yorulmaz, Emel Yantir, Eren Gunduz, Ertugrul Colak

**Affiliations:** 1 Department of Internal Medicine, Faculty of Medicine, Eskisehir Osmangazi University, 26040 Eskisehir, Turkey; 2 Department of Internal Medicine, Division of Endocrinology and Metabolism, Faculty of Medicine, Eskisehir Osmangazi University, 26040 Eskisehir, Turkey; 3 Department of Immunology, Faculty of Medicine, Eskisehir Osmangazi University, 26040 Eskisehir, Turkey; 4 Department of Internal Medicine, Division of Hematology, Faculty of Medicine, Eskisehir Osmangazi University, 26040 Eskisehir, Turkey; 5 Department of Biostatistics, Faculty of Medicine, Eskisehir Osmangazi University, 26040 Eskisehir, Turkey

**Keywords:** subacute thyroiditis, SARS-CoV-2, mRNA vaccines, HLA-A*11, MHC class I, HLA-A*11-B*35-C*04 haplotype

## Abstract

Subacute thyroiditis (SAT) is a thyroid disease associated with viral infections. Its relationship with major histocompatibility complex (MHC) antigens was shown before. SAT cases triggered by different types of severe acute respiratory syndrome coronavirus 2 (SARS-CoV-2) vaccines have been reported. In this study, human leukocyte antigen (HLA) genotypes of 27 SAT patients (13 vaccine-associated (V-SAT) and 14 non-SARS-CoV-2-infection non-vaccine-associated (non-V-SAT)) were compared with those of 362 healthy donors. HLA analyses were performed with low-resolution DNA-based sequence-specific oligonucleotide or sequence-specific primer methods. Statistical analyses were performed using IBM SPSS Statistics 25 and Stata/MP 14.1 with the hapipf function. Allele and haplotype frequencies were estimated by PyPop and gene[RATE] tool programs. The allele frequencies of HLA-A*11, HLA-B*35, and HLA-C*04 were higher in the patient groups. Both the allele frequency of HLA-A*11 and the haplotype frequency of A*11-B*35-C*04 were higher in the V-SAT group. The A*11-B*35-C*04 haplotype, including all three loci of MHC class I genes, is shown to be associated with the disease for the first time, especially in the V-SAT group. This finding will contribute to a better understanding of the etiopathogenesis of vaccine-associated SAT and the role of HLA genotypes in the functioning mechanisms of the SARS-CoV-2 vaccines.

## 1. Introduction

Subacute thyroiditis (SAT) is a disease of the thyroid gland characterized by inflammation and follicle destruction [1]. Like most thyroid diseases, it is more common in women than men. It is the most common cause of painful thyrotoxicosis and accounts for approximately 5% of all thyroid diseases [1,2].

The disease is generally accepted to be triggered by the effect of various viral infections [1]. Since it usually occurs after 2–8 weeks from the active phase of the viral infection, it is classified as subacute [2]. There is a genetic predisposition to SAT, which was shown to be associated with major histocompatibility complex (MHC) antigens [2]. The etiology of SAT cases reported in the literature is not limited to viral infections. There are also cases of SAT associated with viral vaccines like seasonal influenza, H1N1, and hepatitis B viruses [3,4,5]. Starting from the early stages of the coronavirus disease 2019 (COVID-19) pandemic, SAT cases triggered by the severe acute respiratory syndrome coronavirus 2 (SARS-CoV-2) have been described [6,7,8]. Many cases of vaccine-associated SAT triggered by different COVID-19 vaccines have also been presented after the initiation of mass vaccination [9,10,11,12].

SAT is usually a self-limiting disease and mostly results in complete recovery. Sometimes recurrences can be observed [1]. Whether re-exposure to the triggering viral antigen or genetic susceptibilities causes recurrent attacks is not clear yet since the disease is rare, and recurrences are rarer. Permanent hypothyroidism is another outcome of SAT. It is the consequence of healing with fibrosis in the thyroid tissue after the active phase of the disease.

The genetic predisposition associated with MHC antigens in patients developing SAT was first shown by Nyulassy et al. with the demonstration of human leukocyte antigen (HLA)-Bw35 positivity in Caucasian SAT patients [13]. After that, Aiginger et al. published that HLA-Cw4, as well as HLA-Bw35, increases the risk of SAT [14]. Later studies have shown a predominantly association with HLA-Bw35 in both Caucasians and Asians. Articles have also been published showing that HLA-B67, HLA-Cw3, HLA-Dw1, and HLA-DRw8 may also be weakly associated with SAT in some populations [15,16,17]. 

In a recently published study by Stasiak et al. with a large group of patients and controls, HLA-B*18:01, HLA-DRB1*01, and HLA-C*04:01 were reported to be associated with SAT besides the strong association of HLA-B*35 [18]. A small number of familial case reports revealed some alleles being associated with SAT, but it could not be fully demonstrated in studies conducted with large patient groups.

The aim of the current study is to investigate the HLA genotypes that may have a role in the development of SAT in cases associated with the COVID-19 vaccines and cases that are not associated with COVID-19 infection or vaccines and to reveal if there is a different disease-associated HLA allele, a haplotype, or both in the patients.

## 2. Materials and Methods

### 2.1. Study Population and Design

The current study is a case–control study conducted in a single center. The study population consisted of 40 consecutive patients diagnosed with SAT between March 2019 and December 2021 in the Endocrinology and Internal Medicine outpatient clinics of Eskisehir Osmangazi University Faculty of Medicine Training and Research Hospital, which is a tertiary care center. The control group consisted of 362 unrelated healthy donors over the age of 18, whose HLA A, B, C, DRB1, and DQB1 loci were studied with low-resolution DNA-based sequence-specific oligonucleotide (PCR-SSO) or sequence-specific primer (PCR-SSP) methods in Eskisehir Osmangazi University Tissue Typing Laboratory, between 2001 and 2022, for solid organ or bone marrow donation. 

Since patients followed in remission were included, data on diagnosis and treatment were obtained from file records. SAT diagnosis was performed according to the clinical, laboratory, and imaging findings. Clinical findings included anterior neck pain, tenderness in the thyroid lodge, tachycardia, weight loss, and fever. Laboratory findings included thyrotoxicosis, elevated erythrocyte sedimentation rate (ESR), and/or C-reactive protein (CRP) without the signs of active infectious disease. Imaging findings included normal-sized or enlarged thyroid gland with diffusely or focally hypoechogenic areas in the ultrasonographic examination, low blood flow in color Doppler, and/or low uptake in radioiodine or technetium imaging. Patients were followed up initially every 15 days and then monthly in the active phase. After remission (defined biochemically as euthyroidism and return to normal in acute-phase reactants with clinical improvement and the treatment discontinuation if given), follow-up visits were continued every 3–6 months. The treatment modalities, whether patients achieved remission, relapse status, and permanent hypothyroidism were reviewed from the file records. Since the diagnosis of SAT was not supported histopathologically in our patients, HLA analyses were performed not at the time of diagnosis, but after remission, with the confirmation of the diagnosis clinically, considering the cost-effectivity. Patients who did not attend follow-up visits regularly, refused the HLA analysis, or had HLA analysis but did not give consent for use in the study were excluded. Afterward, the patients were divided into groups according to the etiology: cases associated with the SARS-CoV-2 vaccine (V-SAT), cases associated with COVID-19 infection, and cases not associated with COVID-19 infection or vaccines (non-V-SAT). V-SAT was defined as having no recent COVID-19 infection or no history of contact with a positive case, a negative SARS-CoV-2 real-time polymerase chain reaction (RT-PCR) test, and a temporal relationship between SARS-CoV-2 vaccination and SAT clinic (a history of vaccination within the last month). Among the cases diagnosed with SAT during the COVID-19 pandemic, cases in which COVID-19 infection could not be ruled out in the etiology were excluded. SAT patients associated with COVID-19 infection were also excluded because they were insufficient in number to form a separate group. Overall, 27 patients were included in the study.

### 2.2. HLA Typing

DNA was isolated from peripheral blood, which was taken into tubes with K_3_ EDTA using an automated system (EZ1 DNA Blood Kit 200 µL, Qiagen, Hilden, Germany) according to the manufacturer’s instructions. DNA concentrations were measured using the QIAxpert System (QIAGEN GmbH, Hilden, Germany). DNA samples purified to an A260/A280 ratio between 1.65 and 1.80 with a final concentration of 200 ng/µL were amplified using the PCR method (Labcycler SensoQues, GmbH, Göttingen, Germany). The HLA class I (HLA-A, HLA-B, HLA-C) and HLA class II (HLA-DRB1, HLA-DQB1) alleles of patients were typed by PCR-SSO using the LIFECODES HLA Typing Kit (Immucor, Germany) or PCR-SSP method using the Olerup SSP Typing Kits (Stockholm, Sweden) with analysis carried out on the Luminex 100/200 system. HLA alleles were analyzed using MATCH IT! DNA Version 1.3 software (Immucor GTI Diagnostics, Bonn, Germany). The IMGT/HLA database Version 3.43 was used as a reference.

### 2.3. Statistical Analysis

Pearson chi-square, Pearson chi-square exact test, Yates’s chi-square, and Fisher’s exact test were used to determine the relationship between the allele frequencies of the patient and control groups. In addition, logistic regression analysis was used to calculate odds ratios (ORs) and 95% confidence intervals (CIs) for determining the relationship between the presence of specific alleles and the risk of the disease. All analyses were performed using IBM SPSS Statistics 25 and Stata/MP 14.1 with the hapipf function. A *p* value less than 0.05 was accepted as a significance level. 

HLA data were analyzed to estimate linkage disequilibrium (LD), Hardy–Weinberg equilibrium (HWE) proportions, homozygosity test of neutrality, and allele and haplotype frequencies. Allele and haplotype frequencies were estimated by resolving phase and allelic ambiguities using the expectation–maximization (EM) algorithm, both implemented in Python for population genomics (PyPop) Version 0.7.0, The University of California, Berkeley and gene[RATE] tools [19,20]. Slatkin’s implementation of the Ewens–Watterson (EW) homozygosity test of neutrality was performed in PyPop Version 0.7.0. 

### 2.4. Ethical Issues

The study was approved by the Eskisehir Osmangazi University Ethics Committee (Approval No. 68, dated 26 April 2022). The study was carried out in accordance with the statement of the Helsinki Declaration. Informed consent was obtained from each participant. 

## 3. Results

The patient and control groups consisted of individuals from different regions of the country. Among the patient group, 17/27 (63%) were women, 10/27 (37%) were men, and the mean age was 44.77 ± 10.58 years. All patients included had their first SAT attack, and no recurrence was observed in any of the patients during the study period. Seven of the patients were given steroid treatment from another health facility and then applied to our center for the continuation of follow-up. Since our treatment approach is primarily non-steroidal anti-inflammatory drug (NSAID) treatment, NSAIDs were administered in these patients, steroid treatment was discontinued by tapering, and no clinical worsening was observed. The other 20 patients were diagnosed in our center and treated with NSAIDs. Since aminotransferase elevation developed in one patient with the treatment, NSAID was discontinued, and remission was achieved with acetylsalicylic acid (ASA) treatment. Remission was achieved with NSAIDs in the other 26 patients. The number of patients who developed permanent hypothyroidism and received levothyroxine replacement therapy was eight (29.6% of all patients). 

There were 13 cases of vaccine-associated SAT (V-SAT) in the patient group, of which the clinical features of 6 were published previously in our 11-case vaccine-associated SAT article [10]. The other seven V-SAT cases in the current study were diagnosed after the submission of this article and are currently under follow-up in remission. Among V-SAT cases, 8/13 (62%) were women, and 5/13 (38%) were men. The mean age of the patients was 44.92 ± 12.35. SAT had occurred after administration of BNT162b2 Pfizer/BioNTech (Comirnaty) COVID-19 mRNA vaccine in 9/13 (70%), Coronavac inactivated SARS-CoV-2 vaccine in 2/13 (15%), and BNT162b2 administered after two doses of Coronavac in 2/13 (15%) of the patients. Three V-SAT patients were diagnosed in another health facility and administered steroid treatment. These patients were switched to NSAID therapy in our center during follow-up. In one of these patients, permanent hypothyroidism was developed, and levothyroxine replacement therapy was administered. In addition, two more patients had developed permanent hypothyroidism and the need for levothyroxine replacement therapy (the permanent hypothyroidism rate in the V-SAT group was 23%). 

When the HLA phenotypes of the patient group (V-SAT and non-V-SAT groups both) and the control group were compared, it was found that positivity rates for HLA-A*11, HLA-B*35, and HLA-C*04 alleles were higher in the patient group. These alleles were found to be related to increased risk with odds ratios of 2.8, 23.7, and 10.9, respectively (Table 1). There was no homozygosity for these alleles at any of the patient group’s HLA-A, B, and C loci. The HLA-B*35 allele was present in 92.6% (25/27) of the patient group and 34.5% (125/362) of the control group. 

The HLA-B*18 allele, which has been shown to be associated with SAT, was observed in 18.5% (5/27) of the patient group and 9.6% (35/362) of the control group, and an OR of 2.1 (95% CI 0.757–5.958) was found; a statistically significant relationship could not be established. However, two patients in the V-SAT group, who were negative for HLA-B*35, were both HLA-B*18-positive. The HLA-B*67 allele was not observed in any of the patients, as in most studies with other Caucasian SAT patients. No class II HLA allele types that increased SAT risk were found in HLA-DR and HLA-DQ loci in our study. 

When V-SAT cases were analyzed and compared with the control group, the presence of HLA-A*11, HLA-B*35, and HLA-C*04 alleles was found to be related to increased risk (Table 2). 

The HLA-A*11 allele, which has not been shown to be associated with SAT before, being present together with HLA-B*35 and HLA-C*04 alleles, was found to be related to a 6.31-fold (95% CI 2.379–16.782) increase in the risk of SAT in all patients in our study. When the V-SAT and non-V-SAT groups were compared with the control group in terms of the presence of the A*11-B*35-C*04 haplotype, this particular haplotype was found to be associated with a higher risk in the V-SAT group (Table 3).

When the allele frequencies (AFs) were evaluated, HLA-A*11 was the second most common allele at the A locus in the V-SAT group with an AF of 0.192 and the sixth in the non-V-SAT group with an AF of 0.107. In the control group, it was the fifth most common allele with an AF of 0.071. HLA-B*35 was the most common allele at the B locus in both patient groups (AF = 0.4230, 0.5000, respectively). Similarly, HLA-C*04 was the most common allele at the C locus in both groups (AF = 0.3461, 0.5000, respectively). In the control group, HLA-B*35 (AF = 0.1989) was the most common allele at the B locus also, and HLA-C*04 (AF = 0.2030) was the second most common allele at the C locus. A brief list of the most common alleles is presented in Table 4, and a detailed list is provided in Supplementary Table S1.

Looking at the haplotype frequencies (HFs), while the A*11-B*35-C*04 haplotype was the most common in the V-SAT group with an HF of 0.153, it was not among the most common haplotypes in the non-V-SAT group. In the control group, the A*11-B*35-C*04 haplotype was the ninth most common haplotype with an HF of 0.018. A list of the 10 most common haplotypes in the patient and control groups is presented in Table 5.

Slatkin’s implementation of the EW homozygosity test of neutrality analysis and Weinberg equilibrium and global linkage disequilibrium results for patient and control groups are provided in Supplementary Tables S2 and S3. 

## 4. Discussion

The current study aimed to investigate the HLA genotypes of SAT cases associated with the COVID-19 vaccines and compare them with the HLA genotypes of cases not associated with COVID-19 infection or vaccines. The allele frequency of HLA-A*11 and the haplotype frequency of A*11-B*35-C*04 were higher in the V-SAT group, whereas the allele frequencies of HLA-B*35 and HLA-C*04 were higher in the non-V-SAT group. The frequencies of all three alleles and the A*11-B*35-C*04 haplotype were higher in the V-SAT and non-V-SAT groups than in the control group. 

SAT is an immunological disease of the thyroid gland, which is thought to be triggered by viral infections. Thyroid autoimmunity is considered not to have a primary role in SAT [2]. No specific autoantibodies for SAT, similar to those in other autoimmune thyroid diseases, have been demonstrated so far. Cellular immune response rather than humoral immunity plays an essential role in SAT. It is thought that peptide antigens that occur with tissue damage caused by viral infections are recognized by macrophages through MHC class I and presented to cytotoxic T cells. The activation of the CD8+ T cells causes damage to the thyroid tissue [2]. 

SAT has been demonstrated to be associated with various human leukocyte antigens, the best known of which is HLA-B*35. Research aiming to reveal whether different HLA genotypes may also cause a predisposition to SAT in some individuals continues. 

In our study, the HLA genotype data of 27 SAT patients were compared with those of 362 healthy donors, and the presence of HLA-A*11, HLA-B*35, and HLA-C*04 alleles was found to be associated with SAT. The relationship between HLA-B*35 and SAT has been well demonstrated in many studies in the literature. [13,14,15,21]. The relationship between HLA-C*04 and SAT was first observed by Bech et al. and presented in a symposium [22]. It was published in the article by Aiginger et al. Linkage disequilibrium between HLA-B*35 and HLA-C*04 was also highlighted in this article [14]. In the recent study by Stasiak et al., the relationship between SAT and HLA-B*35 and HLA-C*04 was reported [18]. 

The relationship of the HLA-A*11 allele with SAT, which was not mentioned in previous publications, was shown for the first time in this study. The HLA-B*35 and HLA-C*04 alleles, which were shown to be associated with SAT clearly, were present in all 14 patients (100%) in the non-V-SAT group. These two alleles were found in higher frequencies also in V-SAT patients, compared to the control group, but not in the entire group like non-V-SAT. However, it was found that the presence of HLA-A*11 is related to a 4.18-fold increase in V-SAT risk. The presence of the A*11-B*35-C*04 haplotype was also more frequent in V-SAT patients, and it was found to be related to an 8-fold increase in risk, which is higher than the 4.9-fold increase found for the non-V-SAT group. Although the number of positive patients for the common alleles HLA-B*35 and HLA-C*04 in the V-SAT group is lower than that in the non-V-SAT group, the HLA-A*11 allele and the A*11-B*35-C*04 haplotype, the association of which with SAT we have shown for the first time, are more common in V-SAT cases. This is one of the most important findings of our study. 

In a study conducted in our country with unrelated blood donors, the HF of A*11-B*35-C*04 was reported as 0.013 [23]. In our study, the HF of A*11-B*35-C*04 was 0.153 in the V-SAT group and 0.018 in the control group. Although our control group has a higher HF than the general population, we still found significant results, which show the importance of the relationship between the A*11-B*35-C*04 haplotype and vaccine-associated SAT.

In a recently published study conducted in our country, the relationship with the HLA genotype was evaluated in patients developing SAT after SARS-CoV-2 vaccination, and homozygosity for HLA-B*35 and HLA-C*04 alleles was found to be associated with worse thyrotoxicosis and a greater inflammatory reaction [24]. Both in this study and our study, the relationship between the HLA-B*18 allele and SAT could not be demonstrated. However, two patients in the V-SAT group who were HLA-B*35- and HLA-C*04-negative were found to be HLA-B*18-positive. It was noted that one of these patients carried HLA-A*11 and HLA-A*02 alleles in the A locus, and the other had homozygous HLA-A*02. We also observed whether the presence of HLA-B*35 or HLA-B*18 alleles covers all patients, as previously stated by Stasiak et al. [25]. 

In our patient group, HLA-B*35 and HLA-B*18 alleles were detected together in 3/27 (11%) patients, one of whom was in the V-SAT group. In previous publications, recurrence was considered high-risk for this genotype but has not been observed so far in these patients [26]. Recurrent SAT can be defined as SAT that occurs after the complete resolution of the initial attack. Although there is no standard definition for the distinction between recurrence that occurs months or even years after full recovery and steroid dependence in most of the published recurrence studies, the recurrence rates and durations in long-term follow-up have been reported in different series as 4% at 6–21 years and 1.6% at 13.6 ± 5.6 years [27,28]. Considering that the patients included in our study were in a follow-up period of at most three years, and recurrence can be seen even after many years, it is difficult to reach a definite conclusion about the recurrence rate in our patients. A recently published meta-analysis showed that patients treated with glucocorticoids had a 1.84-fold higher risk of recurrence than patients treated with NSAIDs [29]. In a study conducted in our country by Sencar et al., the recurrence rates were found to be significantly higher in patients treated with steroids than those treated with NSAIDs. However, the authors stated that in multivariate regression analyses, steroid treatment was not found to be an independent risk factor for recurrence [30]. In another study performed in our country and published very recently, recurrence was observed in 12 of 137 patients during a 12-year follow-up period, and the risk of recurrence was found to be significantly higher in patients taking steroids than in those taking NSAIDs (OR 23.003, 95% CI 1.828–289.490, *p* = 0.015) [31]. We expect to experience low recurrence rates in the long term, as NSAIDs are the predominant treatment used in our cohort. However, in one of the previously published recurrence studies, the HLA-A*11 allele, the relationship of which with SAT we first showed in our study, was present in one of the three patients reported by Yamamoto et al. [32]. Therefore, perhaps the presence of this allele should be a clue to follow these patients more carefully in terms of recurrence.

In our study, permanent hypothyroidism was observed in 8/27 (29.6%) patients, three of whom were in the V-SAT group. The HLA-A*11 allele was present in three of these patients (two in the V-SAT group and one in the non-V-SAT group), and the A*11-B*35-C*04 haplotype in two of them (one in the V-SAT group and the other in the non-V-SAT group). Sencar et al. demonstrated that anti-thyroid peroxidase antibody positivity and treatment with NSAIDs were the significant variables determining the risk of permanent hypothyroidism [30]. No difference was observed in our patients that developed permanent hypothyroidism in terms of those aspects. Due to the small number of patients with permanent hypothyroidism in our study, it is not possible to comment on whether there is a relationship between this condition and the HLA genotype. 

Aside from HLA-B*35, the relationship of which with SAT is already well known, HLA-A*01 and HLA-A*03 alleles have also been reported in vaccine-associated cases [24,33]. No significant data could be obtained in our study on these alleles.

Peptide antigens emerging with different viral infections or vaccines, either caused by the tissue damage of the virus itself or by antiviral antibodies, may cause SAT by showing homology with different HLA genotypes. The fact that the HLA genotypes associated with SAT differ in diverse populations supports this. The difference in findings between studies conducted so far may also be related to the small sample sizes since SAT is an uncommon disease. 

Although the A*11-B*35-C*04 haplotype was observed phenotypically in 4/13 (30.8%) and 3/14 (21.4%) of the patients in V-SAT and non-V-SAT groups, respectively, when HFs were calculated, it was the most common haplotype in V-SAT, while it was not even among the top 10 most common haplotypes in non-V-SAT. The programs used in the analyses are estimation programs, and to determine the exact haplotype frequencies, real HLA haplotype analyses of the subjects’ parents should also be performed. Although the need for achieving this seems like a limitation, one of our study’s strengths is that we performed data analysis using two different programs that are frequently used among various statistical programs accepted in the literature on population genetics. The low number of patients in the groups is the main limitation of this study and is partly due to the retrospective design. In addition, SAT being a rare disease and SARS-CoV-2 vaccine-associated SAT being a relatively new entity are the other factors contributing to the small number of patients. The size of the control group is one of the strengths of this study. In addition, its composition of unrelated individuals made it possible to make comparisons by creating an HLA pool that better reflects the general population without repeating specific genotypic patterns. Allele and haplotype frequencies were calculated in our study, distinct from the previous research. In this respect, providing evaluation with population genetics methods is another strong side to express. Multicenter studies are needed to increase the number of patients and to obtain more precise information on the subject.

## 5. Conclusions

In conclusion, the HLA-A*11 allele has an important role in all SAT patients, along with the undeniable effects of the HLA-B*35 and HLA-C*04 alleles. In vaccine-associated SAT cases, although the positivity rates for HLA-B*35 and HLA-C*04 alleles in our cohort were lower than those of the non-vaccine group, the positivity rates for the HLA-A*11 allele and the A*11-B*35-C*04 haplotype were higher. This reveals the importance of this molecule, the relationship of which with SAT is shown for the first time, especially in vaccine-associated cases. 

Considering the importance of the CD8+ T cells and MHC class I’s role in antigen presentation to these cells in the pathogenesis of SAT, the A*11-B*35-C*04 haplotype including all three loci of MHC class I genes, which is found to be associated with the disease for the first time, especially in the V-SAT group in our study, will contribute both to a better understanding of the etiopathogenesis of SARS-CoV-2 vaccine-associated SAT and the role of HLA genotypes in the functioning mechanisms of the COVID-19 vaccines. 

## Figures and Tables

**Table 1:** The numbers and percentages of positive and negative individuals in terms of specific alleles in patient and control groups and the calculated odds ratios and 95% confidence intervals (CIs) for the risk with these alleles.

		Patients *n* (%)	Controls *n* (%)	*p* Value	OR	95% CI
HLA-A*11	+	8 (29.6%)	47(13%)	0.038 *	2.822	1.169–6.811
	−	19 (70.4%)	315 (87%)			
HLA-B*35	+	25 (92.6%)	125 (34.5%)	<0.001 **	23.7	5.523–101.692
	−	2 (7.4%)	237 (65.5%)			
HLA-C*04	+	23 (85.2%)	125 (34.5%)	<0.001 **	10.902	3.689–32.221
	−	4 (14.8%)	237 (65.5%)			

*: Fisher’s exact test, **: Yates’ chi-square, OR: odds ratio, CI: confidence interval.

**Table 2:** The numbers and percentages of positive and negative individuals in terms of specific alleles in V-SAT, non-V-SAT, and control groups and the calculated odds ratios for the risk with these alleles in V-SAT vs. control groups.

		Non-V-SAT *n* (%)	V-SAT *n* (%)	Controls *n* (%)	*p* Value	V-SAT vs. Controls	
						OR	95% CI
HLA-A*11	+	3 a,b (21.4%)	5 b (38.5%)	47 a (13.0%)	0.025 *	4.189	1.315–13.344
	−	11 a,b (78.6%)	8 b (61.5%)	315 a (87.0%)			
HLA-B*35	+	14 a (100%)	11 a (84.6%)	125 b (65.5%)	<0.001 *	10.428	2.276–47.781
	−	0 a (0%)	2 a (15.4%)	237 b (65.5%)			
HLA-C*04	+	14 a (100%)	9 a (69.2%)	125 b (34.5%)	<0.001 *	4.266	1.288–14.129
	−	0 a (0%)	4 a (30.8%)	237 b (65.5%)			

*: Exact test with Monte Carlo simulation method, OR: odds ratio, CI: confidence interval, V-SAT: vaccine-associated subacute thyroiditis, Non-V-SAT: subacute thyroiditis due to other causes. a, b: Each subscript with the same letter denotes that column proportions do not differ significantly from each other at the 0.05 significance level.

**Table 3:** The numbers and percentages of positive and negative individuals in terms of the A*11-B*35-C*04 haplotype in V-SAT, non-V-SAT, and control groups and the calculated odds ratios for the risk with this haplotype.

		Non-V-SAT *n* (%)	V-SAT *n* (%)	All Patients *n* (%)	Controls *n* (%)	*p* Value	Non-V-SAT vs. Controls		V-SAT vs. Controls		All Patients vs. Controls	
							OR	95%CI	OR	95% CI	OR	95% CI
Haplotype	+	3 a (21.4%)	4 a (30.8%)	7 a (25.9%)	19 b (5.2%)	0.002 *	4.923	1.267–19.137	8.023	2.264–28.432	6.318	2.379–16.782
	−	11 a (78.6%)	9 a (69.2%)	20 a (74.1%)	343 b (94.8%)							

*: Exact test with Monte Carlo simulation method, OR: odds ratio, CI: confidence interval, V-SAT: vaccine-associated subacute thyroiditis, Non-V-SAT: subacute thyroiditis due to other causes. a, b: Each subscript with the same letter denotes that column proportions do not differ significantly from each other at the 0.05 significance level. All pairwise comparisons are tested with Bonferroni adjustment by using column proportions.

**Table 4:** MHC class I allele frequencies for groups.

	V-SAT		Non-V-SAT		Controls	
**HLA-A**		**AF**		**AF**		**AF**
	A*02	0.2307	A*02	0.2500	A*02	0.2486
	A*11	**0.1923**	A*03	0.1428	A*24	0.1409
	A*24	0.1538	A*24	0.1428	A*03	0.1229
	A*03	0.1153	A*01	0.1071	A*01	0.1008
	A*01	0.0769	A*11	**0.1071**	A*11	**0.0718**
**HLA-B**						
	B*35	**0.4230**	B*35	**0.5000**	B*35	**0.1989**
	B*18	0.1153	B*51	0.1071	B*51	0.1229
	B*38	0.0769	B*07	0.0714	B*44	0.0760
	B*07	0.0384	B*18	0.0714	B*07	0.0691
	B*15	0.0384	B*27	0.0714	B*49	0.0580
**HLA-C**						
	C*04	**0.3461**	C*04	**0.5000**	C*07	0.2182
	C*07	0.2307	C*12	0.1071	C*04	**0.2030**
	C*12	0.1538	C*02	0.0714	C*12	0.1298
	C*08	0.0769	C*07	0.0714	C*06	0.1022
	C*01	0.0384	C*15	0.0714	C*15	0.0677

AF: allele frequency. The frequencies of HLA-A*11, HLA-B*35, and HLA-C*04 alleles are shown in bold.

**Table 5:** ABC haplotype frequencies for groups.

V-SAT		Non-V-SAT		Controls	
	HF		HF		HF
**A11-B35-C04**	**0.1538**	A02-B35-C04	0.2143	A02-B35-C04	0.0397
A01-B35-C04	0.0769	A03-B35-C04	0.1071	A24-B35-C04	0.0333
A02-B18-C07	0.0769	A24-B35-C04	0.0714	A03-B35-C04	0.0291
A03-B35-C04	0.0769	A25-B18-C12	0.0357	A23-B49-C07	0.0274
A26-B35-C12	0.0385	A11-B07-C07	0.0357	A02-B51-C15	0.0240
A29-B47-C07	0.0385	A02-B27-C02	0.0357	A03-B07-C07	0.0238
A24-B35-C04	0.0385	A68-B51-C15	0.0357	A01-B35-C04	0.0213
A24-B40-C03	0.0385	A24-B73-C15	0.0357	A02-B07-C07	0.0194
A02-B44-C05	0.0385	A01-B50-C06	0.0357	**A11-B35-C04**	**0.0185**
A24-B45-C06	0.0385	A01-B35-C04	0.0357	A30-B13-C06	0.0159

HF: haplotype frequency. The frequencies of A*11-B*35-C*04 haplotype are shown in bold.

## Data Availability

The data presented in this study are available on request from the corresponding author. The data are not publicly available due to ethical reasons.

## Supplementary Materials

The following supporting information can be downloaded at: https://www.mdpi.com/article/10.3390/vaccines10121986/s1; Table S1: MHC class I allele frequencies for groups; Table S2: Slatkin’s implementation of EW homozygosity test of neutrality analysis; Table S3: Weinberg equilibrium and global linkage disequilibrium.
