# Supplementary material for: A Novel Finding of an HLA Allele’s and a Haplotype’s Relationship with SARS-CoV-2 Vaccine-Associated Subacute Thyroiditis
Source: Vaccines (Basel). 2022 Nov 23;10(12):1986. doi: 10.3390/vaccines10121986 (PMC9788374; doi:10.3390/vaccines10121986)
Supplement: Supplementary file 1 [file vaccines-10-01986-s001.zip › vaccines-1910900-supplementary.pdf]

Scheme 1. MHC Class I allele frequencies for groups.

	V-SAT		non-V-SAT		Controls	
**HLA-A**		**AF**		**AF**		**AF**
	A*02	0.2307	A*02	0.2500	A*02	0.2486
	**A*11**	**0.1923**	A*03	0.1428	A*24	0.1409
	A*24	0.1538	A*24	0.1428	A*03	0.1229
	A*03	0.1153	A*01	0.1071	A*01	0.1008
	A*01	0.0769	**A*11**	**0.1071**	**A*11**	**0.0718**
	A*26	0.0769	A*68	0.0714	A*26	0.0566
	A*29	0.0384	A*25	0.0357	A*32	0.0470
	A*30	0.0384	A*26	0.0357	A*23	0.0442
	A*32	0.0384	A*31	0.0357	A*30	0.0442
	A*68	0.0384	A*32	0.0357	A*68	0.0359
			A*69	0.0357		
**HLA-B**						
	**B*35**	**0.4230**	**B*35**	**0.5000**	**B*35**	**0.1989**
	B*18	0.1153	B*51	0.1071	B*51	0.1229
	B*38	0.0769	B*07	0.0714	B*44	0.0760
	B*07	0.0384	B*18	0.0714	B*07	0.0691
	B*15	0.0384	B*27	0.0714	B*49	0.0580
	B*40	0.0384	B*38	0.0357	B*18	0.0511
	B*44	0.0384	B*40	0.0357	B*13	0.0428
	B*45	0.0384	B*50	0.0357	B*38	0.0401
	B*47	0.0384	B*56	0.0357	B*15	0.0373
	B*48	0.0384	B*73	0.0357	B*40	0.0345
	B*51	0.0384				
	B*55	0.0384				
	B*58	0.0384				
**HLA-C**						
	**C*04**	**0.3461**	**C*04**	**0.5000**	C*07	0.2182
	C*07	0.2307	C*12	0.1071	**C*04**	**0.2030**
	C*12	0.1538	C*02	0.0714	C*12	0.1298
	C*08	0.0769	C*07	0.0714	C*06	0.1022
	C*01	0.0384	C*15	0.0714	C*15	0.0677
	C*03	0.0384	C*01	0.0357	C*03	0.0483
	C*05	0.0384	C*03	0.0357	C*01	0.0414
	C*06	0.0384	C*06	0.0357	C*16	0.0414
	C*14	0.0384	C*14	0.0357	C*14	0.0401
			C*16	0.0357	C*02	0.0345

AF: allele frequency. The frequencies of HLA-A*11, HLA-B*35, and HLA-C*04 alleles are shown in bold.

Scheme 2. Slatkin's implementation of EW homozygosity test of neutrality analysis.

	Obs	Exp	Variance	Fnd	p
			**Patients**		
**A**	0.1365	0.1696	0.0024	-0.6749	0.2557
**B**	0.2407	0.1204	0.0010	3.8743	0.9923
**C**	0.2305	0.1865	0.0032	0.7811	0.8316
**DRB1**	0.1427	0.1696	0.0024	-0.5490	0.3248
**DQB1**	0.3169	0.4460	0.0198	-0.9187	0.1754
			**Controls**		
**A**	0.1243	0.2423	0.0089	-1.2489	0.0202
**B**	0.0837	0.1534	0.0030	-1.2631	0.0160
**C**	0.1312	0.2887	0.0128	-1.3916	0.0040
**DRB1**	0.1197	0.3079	0.0142	-1.5804	0.0010
**DQB1**	0.2723	0.5963	0.0371	-1.6827	0.0099

Obs: Observed F, Exp: Expected F, Variance: Variance in F, Fnd: Normalized deviate of F.

Scheme 3. Weinberg Equilibrium and Global Linkage Disequilibrium.

Patients	A	B	C	DR	DQ	Controls	A	B	C	DR	DQ
**A**	1.0000	1.0000	1.0000	1.0000	0.9335	**A**	0.0167	0.0001	0.0000	0.4381	0.9721
**B**	1.0000	1.0000	0.9999	1.0000	0.9992	**B**	0.0099	0.0000	0.0000	0.0000	0.0000
**C**	1.0000	0.1287	1.0000	1.0000	0.9998	**C**	0.0099	0.0099	0.0026	0.0000	0.0007
**DR**	1.0000	1.0000	1.0000	1.0000	0.0097	**DR**	0.0693	0.0099	0.0099	0.0005	0.0000
**DQ**	0.2970	0.7129	0.9505	0.0099	1.0000	**DQ**	0.7228	0.0099	0.0099	0.0099	0.0008

The diagonal contains the p values for the Hardy–Weinberg equilibrium test, the upper triangular matrix those for the parametric global LD test, and the lower triangular matrix those for the permutation non–parametric global LD test.
